# #influenced! The impact of social media influencing on self-esteem and the role of social comparison and resilience

**DOI:** 10.3389/fpsyg.2023.1216195

**Published:** 2023-10-04

**Authors:** Lale Rüther, Josephine Jahn, Tamara Marksteiner

**Affiliations:** Department of Psychology, University of Mannheim, Mannheim, Germany

**Keywords:** social media influencers, self-esteem, social comparison, resilience, moderated mediation model, experimental research design

## Abstract

Social media influencers (SMIs) are online personas that acquire significant audiences on social networking sites (SNS) and have become a prevalent part of social media. Previous research indicates potentially detrimental effects of social media use on mental well-being, however, little is known about whether, how, and for whom online comparisons with SMIs lead to adverse psychological effects. In this study, we investigate the impact of positivity-biased images of female SMIs on the state self-esteem of female participants while considering social comparison processes as mediating and individual resilience as moderating factors. Regression analyses showed that acute exposure to positivity-biased SMI images led to upward social comparisons, which in turn predicted lower state self-esteem. Thus, results revealed a significant mediating effect of social comparisons on the association between image type and state self-esteem. However, when observing the direct effect of image type on state self-esteem, we found that the exposure to positivity-biased SMI images unexpectedly led to higher overall levels of state self-esteem relative to the control group. In light of contemporary social comparison literature, subsequent post-hoc analyses suggest that exposure to SMI images in this study may have prompted both contrastive and assimilative upwards comparisons, leading to varying consequences for distinct self-esteem dimensions, ultimately manifesting in the observed suppression effect. Resilience was not found to moderate the proposed associations. Thus, the findings of this study offer new insights into the impact of SMIs on individuals’ self-evaluations online, challenging previous assumptions, and suggest a need for further examination.

## 1. Introduction

Social media plays a central role in modern society, influencing how people access information, find entertainment and construct their identities (Hajli, 2014; Herring and Kapidzic, 2015). Research indicates that social media use can harm psychological well-being due to online social comparisons (Tiggeman and Zaccardo, 2015; Liu and Baumeister, 2016; Verduyn et al., 2017). Frequent users tend to see others as happier and more successful (de Vries et al., 2017; Midgley et al., 2021). This perception is amplified by a *social media positivity bias* (Schreurs and Vandenbosch, 2021), a tendency where individuals selectively present overly positive self-images online. This is particularly evident on platforms where imagery can be used to create a seemingly authentic self-image (Bell, 2019). Previously, social media use has been associated with negative psychological outcomes—mediated by social comparisons—such as lower life satisfaction, increased loneliness, and body-image concerns (Lup et al., 2015; Tiggeman and Zaccardo, 2015; Liu and Baumeister, 2016; Appel et al., 2020; Pedalino and Camerini, 2022). However, studies on the relationship between social media use and self-esteem show mixed results, with some finding negative (Tiggeman and Zaccardo, 2015; Liu and Baumeister, 2016), while others note positive (Gonzales and Hancock, 2011) or non-significant connections (Liu and Baumeister, 2016; Appel et al., 2020).

## 2. Theoretical background and hypotheses

### 2.1. The mediating role of social comparisons

Previous research underscores social comparisons’ role in mediating SNS effects on self-esteem (Tiggeman and Zaccardo, 2015; Krause et al., 2021; Midgley et al., 2021). Instagram’s visual nature and editing features encourage positively biased self-presentation that can drive harmful upwards comparisons, mainly for those feeling inadequate to online ideals, often set by social media influencers (SMIs) (Lup et al., 2015; Schreurs et al., 2022). Referred to as micro-celebrities, SMIs can perpetuate unattainable comparison standards (Schreurs and Vandenbosch, 2021), fostering insecurities among viewers (Gräve, 2017; Chae, 2018; Pedalino and Camerini, 2022). Initial findings link Instagram browsing to body dissatisfaction, particularly among adolescents comparing themselves to influencers (Pedalino and Camerini, 2022). However, a research gap hinders understanding of the impact of social comparisons with SMIs on viewers’ self-esteem (Verduyn et al., 2017; Appel et al., 2020).

**Figure 1:**
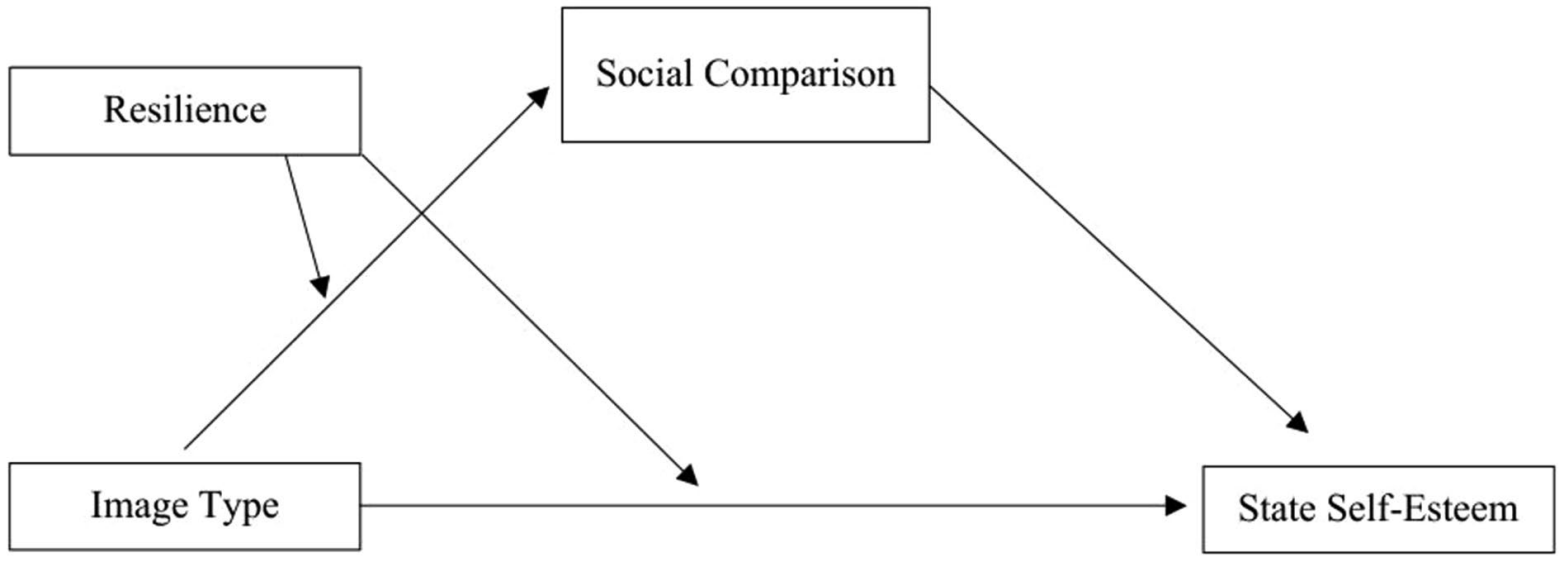
Conceptual model of proposed associations in the present study. Image type was dummy coded (1 = SMI images, 0 = control images). Social comparison was measured using a semantic differential (SSC, Allan and Gilbert, 1995); lower scores on the measure reflect upward comparisons, higher scores reflect downward comparisons. Directions of associations as proposed in Hypotheses 1 to 4.

Existing literature shows parallels in other media contexts, like lower body self-esteem linked to same-gendered models in fashion magazines (Grogan et al., 1996). Moreover, social comparisons with SNS celebrities affect female adolescents’ body image and drive for thinness negatively (Ho et al., 2016). Overall, studies investigating gender differences in self-esteem reveal a gender gap, with women reporting lower self-esteem across cultures and ages (Bleidorn et al., 2016) and a tendency for women to engage more in negative upwards social comparisons (Ho et al., 2016; Valls, 2022). This emphasizes women’s susceptibility to social media-induced upward comparisons. We hypothesize that exposure to positivity-biased SMI images on Instagram lowers female participants’ state self-esteem compared to neutral images of women (Hypothesis 1; Figure 1). We further assume that female individuals viewing SMI posts engage in upward social comparisons, leading to lower self-esteem, with upward comparisons mediating this association (Hypothesis 2, Figure 1).

### 2.2. The moderating role of individual resilience

Resilience, the ability to adapt and rebound from stress and adversity aided by personal, social and situational resources (Windle, 2011), is linked to self-esteem through positive emotions (Benetti and Kambouropoulos, 2006). However, limited research explores individual resilience in social media settings. Bilgin and Taş (2018) found that higher resilience helps coping with negative online experiences, vital since online comparisons are tied to depressive symptoms, loneliness and negative body image (e.g., Tiggeman and Zaccardo, 2015; Ho et al., 2016; Appel et al., 2020). We view upward comparisons with SMIs as aversive experiences that could damage self-esteem and propose that resilience mitigates these effects. We hypothesize that greater resilience helps coping with positivity-biased SMI images, increasing self-esteem and reducing upward comparisons. Consequently, we expect resilience to moderate the link between SMI exposure and state self-esteem (Hypothesis 3) and social comparison (Hypothesis 4).

### 2.3. Gaps in research

Despite a rapid increase in psychological research related to social media, drawing generalizable conclusions remains challenging (Appel et al., 2020). Limited experimental studies hinder establishing causality between social media use and mental well-being (Appel et al., 2020). Meta-analyses by Appel et al. (2020) and Liu and Baumeister (2016) suggest small and inconclusive associations. Overlooking visually centered platforms, past studies primarily focused on Facebook use. However, images have since emerged as the most popular medium of online self-expression (Herring and Kapidzic, 2015), highlighting the need to test the accuracy of previous findings in these social networking environments (Verduyn et al., 2017). This study addresses these gaps, investigating associations between state self-esteem, social comparisons, and resilience after exposure to SMI images on Instagram, using an experimental design.

## 3. Methods

### 3.1. Participants

We recruited 245 university students, who identified as female. After excluding 14 participants for attention check failure or incomplete surveys, the final sample consisted of 231 participants aged 18 to 35 years (*M_age_* = 23.17, *SD* = 3.18).

### 3.2. Design

This experimental study used a between-subjects design, varying the independent variable image type (SMI vs. control images). Self-esteem and social comparison were main dependent variables, with the latter entered as a mediator. Resilience was tested as a moderator for the relationships between image type, self-esteem and social comparison.

### 3.3. Materials

Participants randomly viewed one of two distinct sets of images, each containing 15 images of women. For both groups, images were presented one below the other to simulate the direction of scrolling in an Instagram feed. Participants in the SMI group viewed 15 images of female influencers, priorly selected based on two open access surveys listing the most-followed German influencers on Instagram in 2019 (InfluencerDB, 2020a,b). The images were selected based on the depiction of staged situations in which the influencer posed by angling their face or body toward the camera. Five images featured designer brand items, five others exhibited exotic vacation locations, and five images highlighted the influencer’s physical appearance (e.g., selfie). Each profile name was displayed underneath its designated image. The control image set contained 15 film photographs of women, provided by photographer Giulia Thinnes (https://www.giuliathinnes.com). The images were chosen due to their authentic portrayal of women in offline environments without positivity-biased features. The control image set matched the SMI images in terms of color scheme, the position (e.g., sitting, standing) and perspective on the displayed individuals. Five images displayed the women holding an object (e.g., book, bicycle), five displayed nature in the background (e.g., field, garden) and five more images showed a neutral background (e.g., wall). The photographer’s name was displayed underneath each image.

### 3.4. Measurements

#### 3.4.1. State self-esteem

State self-esteem was measured using a German translation of the State Self-Esteem Scale (SSES; Heatherton and Polivy, 1991). It consists of 20 items assessing short-lived changes in self-esteem using three subscales (i.e., performance-, social-, and appearance- related self-esteem) on a 5-point Likert scale (1 = *does not apply at all* to 5 = *applies completely*). Higher scores on the SSES indicate higher self-esteem (Cronbach’s α = 0.87).

#### 3.4.2. Social comparison

Social comparison was measured using a German translation of the Social Comparison Scale (SCS; Allan and Gilbert, 1995), which assesses self-perception of social rank, perceived attractiveness, and relative social standing in relation to others (Allan and Gilbert, 1995)^1^. This measure was specifically selected due to its capacity to capture state social comparisons and temporary self-evaluations. The SCS measures social comparison in eleven items using a 5-point semantic differential methodology with two bipolar self-descriptive adjectives each (Cronbach’s α = 0.77). Participants were instructed to rate themselves relative to the women in the images they had previously viewed. Lower scores on the SCS reflect feelings of inferiority and low rank self-perception in relation to comparison targets, indicating upwards social comparisons, whereas higher scores indicate feelings of superiority and high rank self-perception, suggesting downwards social comparisons. The middle score (3) represents a neutral self-perception in comparison.

#### 3.4.3. Resilience

Individual resilience was measured using the German Resilience Scale for Adults by Kaiser et al. (2019). The scale assesses resilience according to the current research consensus, conceptualizing resilience as a multilevel construct of protective factors such as personal competencies, support structures and situational factors that determine the temporary ability of coping with stressors and aversive experiences (Windle, 2011 Leipold, 2015). The scale uses a 7-point semantic differential with opposite response alternatives in 33 items and six subscales corresponding to the key dimensions of individual resilience: perception of self (PS), planned future (PF), social competence (SC), structured style (SS), family cohesion (FC) and social resources (SR). To assess the multilevel nature of resilience, all subscales (except for one) were included in the present study. However, the subscales were shortened to reduce participant burden. Respectively, items with item-total-correlation coefficients greater or equal *r_it_* = 0.50 (Döring and Bortz, 2016), were selected from the PS (Items 19, 25, 29), PF (Items 8, 14, 20), FC (Items 10, 16, 27), SR (Items 05, 28, 32), and SC (Items 15, 21) subscales. Items from the SS subscale were not included due to not meeting the item-total-correlation threshold in accordance with recommendation of the scale’s authors (Kaiser et al., 2019). Therefore, participants in the present study answered 14 items from five subscales of the German RSA (Cronbach’s α = 0.80).

#### 3.4.4. Analytical procedure

All analyses were conducted using SPSS Version 29.0. The relationships between image type, social comparison, and state self-esteem were tested in a simple mediation model (Model 4) using Hayes’ SPSS PROCESS macro (Hayes, 2022; IBM Corp, 2022) and the proposed moderating effects of resilience were tested in a moderated mediation model (Model 8) using the same macro.

## 4. Results

### 4.1. Descriptive statistics

Means, standard deviations and Pearson’s correlations on all study variables are displayed in Table 1. Skewness and kurtosis values of the dependent variables were calculated and a normal distribution of the data could be assumed (Table 1). An independent samples *t*-test showed no age differences, *t*(229) = −1.24, *p* = 0.22, between the groups. Unexpectedly, image type and state self-esteem correlated positively, and participants in the SMI group (dummy variable 1) reported slightly higher state self-esteem (*M* = 3.36, *SD* = 0.57) than participants in the control group (dummy variable 0; *M* = 3.23, *SD* = 0.57), *t*(229) = −1.8, *p* = 0.04. However, image type and social comparison correlated negatively, indicating an upwards comparison tendency for the SMI group (*M* = 2.81, *SD* = 0.54), whereas participants in the control group reported a more neutral position (*M* = 3.06, *SD* = 0.39), *t*(229) = 4.10, *p* < 0.001. Social comparison and state self-esteem correlated positively, suggesting that individuals engaging in downwards comparisons also reported higher state self-esteem. Resilience also correlated positively with state self-esteem and social comparison, suggesting that individuals with higher resilience also reported higher state self-esteem as well as a downwards comparison tendency. As expected, the correlation between image type and resilience was non-significant (*p* = 0.12).

**Table 1:** Pearson product–moment correlation coefficients between the independent variable and the dependent variables and variable distributions.

	*M*	*SD*	Skewness	Kurtosis	(1)	(2)	(3)	(4)
1.Image type					1			
2.State self-esteem	3.30	0.58	−0.10	−0.65	0.12*	1		
3.Social comparison	2.93	0.49	−0.40	0.30	−0.26**	0.58**	1	
4.Resilience	3.76	0.53	−0.71	0.18	0.08	0.57**	0.39**	1
Age					0.08	−0.07	0.04	0.07

*N* = 231. The predictor variable was image type, the outcome variables were state self-esteem, social comparison, and resilience. Image type was dummy coded (1 = SMI images, 0 = control images).

*p < 0.05, **p < 0.001.

### 4.2. Mediating effect of social comparison

A simple mediation model was performed to test whether SMI images would predict lower state self-esteem (H1) and whether upwards social comparisons would mediate this association (H2; Table 2). Age was entered as a control variable. The preconditions for regression analysis were met (Field, 2018; Hayes, 2022) and bootstrapping (*n* = 5,000) was used to determine 95% bias-corrected confidence intervals (95% BCa *CI*). The total effect of image type on state self-esteem was non-significant (*p* = 0.09) indicating that the exposure to SMI images did not predict lower state self-esteem in an overall association when direct and indirect effects were considered. Thus, Hypothesis 1 was not supported by the results. The indirect effect of image type on state self-esteem via social comparison was negative (standardized indirect effect = −0.35, SE = 0.09, 95% BCa *CI*: [−0.55, −0.18]) indicating that social comparison mediated the relationship between image type and state self-esteem. Results showed that participants in the SMI group engaged more in upwards comparisons, which in turn related to lower state self-esteem, thus supporting Hypothesis 2. However, the direct effect of image type on state self-esteem was unexpectedly positive (β = 0.58, SE = 0.06, 95% BCa CI [0.21; 0.46]), opposing the observed indirect effect. This pattern of results suggests the occurrence of a suppression effect (MacKinnon et al., 2000), which was probed in a subsequent post-hoc analysis.

**Table 2:** Results from the mediation analysis.

Predictors	Social comparison				State self-esteem			
	*β*	*SE*	*p*	95% CI	*β*	*SE*	*p*	95% CI
Image type	−0.54	0.06	<0.001	[−0.38; −0.14]	0.58	0.06	<0.001	[0.21; 0.45]
Social comparison					0.66	0.06	<0.001	[0.65; 0.9]
Age	0.09	0.01	0.15	[−0.01; 0.03]	−0.03	0.01	0.53	[−0.02; 0.01]
*F*	6.29				39.48			
*R* ^2^	0.08**				0.41**			

*N* = 231. The predictor variable was image type, social comparison was the mediating and state self-esteem the outcome variable. Mediation analysis was conducted using PROCESS Model 4. Image type was dummy coded (1 = SMI images, 0 = control images). Bootstrapping was used for the 95% BCa CIs (*n* = 5,000).*p < 0.05, **p < 0.001.

### 4.3. Post-hoc analysis of the suppression effect

To probe the role of social comparison in the observed suppression effect, we conducted a comparative assessment of regression coefficients and significance levels both prior to and following its inclusion in a regression model. Interestingly, the relationship between image type and self-esteem initially reflected in a linear regression model (b = 0.12, SE = 0.08, *p* = 0.08) exhibited heightened magnitude and significance upon inclusion of social comparison in a multiple linear regression model (b = 0.29, SE = 0.06, *p* < 0.001; MacKinnon et al., 2000). This substantiates the role of social comparison as a possible suppressor variable in this study. To furthermore shed a light on the inconsistency in self-esteem levels across the direct and indirect effects, we analyzed correlations between the different self-esteem subscales and relevant study variables. Social (*r* = 0.12, *p* = 0.04) and performance-related (*r* = 0.17, *p* = 0.005) self-esteem showed weak positive relationships with image type in an overall association, indicating higher self-esteem among those exposed to SMI images. In contrast, appearance-related self-esteem had a non-significant association with image type (*r* = 0.003, *p* = 0.51). Appearance-related self-esteem correlated positively with social comparison (*r* = 0.54, *p* < 0.001), indicating a direct link between upwards comparisons and lower appearance self-esteem. Social (*r* = 0.32, *p* < 0.001) and performance-related (*r* = 0.21, *p* < 0.001) self-esteem also correlated positively with social comparison.

### 4.4. Moderated mediation effects

To examine whether resilience moderated the associations between image type and social comparison (H3) as well as image type and state self-esteem (H4), a moderated mediation model was estimated. Age was entered as a control variable. Results (Table 3) showed statistically non-significant interactions between image type and resilience for both dependent variables social comparison and state self-esteem. Moreover, the index of moderated mediation was non-significant (index: β = 0.05, 95% *BCa CI*: [−0.09, 0.18]). Thus, Hypotheses 3 and 4 were not supported by the results in this study.

**Table 3:** Results from the moderated meditation analysis.

Predictors	Social comparison			95% CI	State self-esteem			95% CI
	*β*	*SE*	*p*		β	*SE*	*p*	
Image-type	−0.53	0.06	<0.001	[−0.4; −0.18]	0.56	0.06	<0.001	[0.14; 0.37]
Resilience	0.34	0.08	<0.001	[0.19; 0.5]	0.43	0.08	<0.001	[0.28; 0.58]
Image type × resilience	0.08	0.11	0.46	[−0.13; 0.29]	−0.08	0.10	0.44	[−0.28; 0.12]
Social comparison					0.6	0.06	<0.001	[0.47; 0.72]
Age	0.02	0.01	0.03	[0.02; 0.04]	0.001	0.01	0.85	[−0.02; 0.02]
*R* ^2^	0.25**				0.51**			
*F*	19.15				47.72			

*N* = 231. Moderated mediation analysis was conducted using PROCESS Model 8. Image type was dummy coded (1 = SMI images, 0 = control images). Bootstrapping was used for the 95% BCa CIs (*n* = 5,000). *p < 0.05, **p < 0.001.

## 5. Discussion

### 5.1. The mediating role of social comparison

Using a simple mediation model, we examined the effects of image type on state self-esteem via social comparison. As hypothesized, social comparison mediated the relationship between image type and state self-esteem. Exposure to SMI images predicted upward comparisons, leading to lower self-esteem. Interestingly, the direct effect contradicted this, revealing a positive relationship between exposure to SMI images and self-esteem. This inconsistent mediation model may seem counterintuitive, but contemporary social comparison research provides insights for interpretation. Central to this discussion is Collins’ Upwards Assimilation Theory (2000), which suggests that individuals who compare themselves to “better-off” others, tend to seek similarities. According to Collins (2000), perceiving to share attributes with a “superior” comparison target can trigger upwards assimilations and subsequently elevate self-worth of the comparing individual. In our study, both participant groups shared notable attributes, like age, gender and nationality, with their comparison targets. However, as hypothesized (Hypothesis 1), only the SMI group viewed their targets as “superior” across various factors, while the control group showed neutral comparisons without a clear indication of either upward or downward direction. In this context, the heightened self-esteem observed in our study can be attributed to upwards assimilation, driven by the impression that participants share attributes with the presented SMIs, whom they viewed as “superior” in various dimensions. Conversely, the absence of upward assimilation among the control group may explain their comparatively lower self-esteem scores. This suggests that the SMI group’s engagement in upwards comparisons fostered assimilative tendencies, temporarily boosting self-esteem.

Further analyses of self-esteem subscales provided valuable insights. Social and performance-related self-esteem correlated positively with image type in an overall association, whereas appearance-related self-esteem showed no significant association. This suggests that the positive direct effect in our study might be driven particularly by social and performance-related self-esteem dimensions. Conversely, upwards comparisons were directly linked to lower appearance self-esteem. As the correlations for social and performance-related self-esteem in this relationship were weaker, it seems that appearance-related self-esteem subscale played a prominent role in determining the negative indirect effect.

An alternative explanation can be drawn from a recent article by Kim et al. (2021), suggesting that emotional contagion, where individuals mirror emotions from Instagram posts, can precede social comparison (Choi and Kim, 2021). According to them, browsing “positive” images on social media can boost positive affect and enhance life satisfaction through emotional contagion. They also suggest that both, contrastive social comparison and emotional contagion, can occur when individuals view positive and upwards comparison-inducing imagery. This perspective could elucidate why SMI images affected self-esteem differently in our mediation model. Positively-biased SMI images might have induced positive affect through emotional contagion, leading to higher self-esteem in social and performance-related dimensions. Nevertheless, participant still engaged in contrastive upwards social comparisons regarding appearance, resulting in lower self-esteem scores in that aspect. Kim et al. (2021) further emphasizes the significance of emotions in online social comparison processes and find similar opposing effects on self-esteem. They discovered that negative emotions (envy and depression) mediated the link between SNS addiction and lower self-esteem, whereas positive emotions (contentment) mediated the relationship between SNS addiction and higher self-esteem. This underscores the need for further research to explore affective responses after self-comparisons with SMIs and their potential impact on self-esteem dimensions.

Expanding on Festinger’s (1954) work, recent social comparison research emphasize that individuals evaluate others not only along vertical dimensions of comparison (e.g., status, agency), but also along horizontal dimensions, considering factors like solidarity or communion (Locke, 2005). This framework suggests that the SMI and control images in our study, might have prompted comparisons along distinct dimensions, potentially contributing to the unexpected results. Horizontal and vertical comparisons have distinct predictors, guiding individuals to evaluate attributes as better or worse (vertical) or as similar or different (horizontal) to themselves (Locke, 2005). Unfortunately, our social comparison measure could not differentiate these dimensions, limiting result interpretation. However, it’s plausible that the images presented to the SMI group, rich in agentic attributes and status-related symbols (e.g., designer items), primed vertical comparisons. In contrast, the control group’s images, lacking these attributes, conveyed a larger sense of relatability and similarity, facilitating horizontal comparisons (Locke, 2005).

Considering the various theoretical frameworks discussed, we conclude that exposure to SMI images impacted distinct self-esteem dimensions differently. This could be attributed to various social comparison mechanisms, including contrasting and assimilative upwards comparisons as well as emotional contagion processes and affective responses. Participants potentially simultaneously engaged in contrastive upwards comparisons with SMIs, associated with lower appearance-related self-esteem, leading to a negative indirect effect but also assimilative upwards comparisons or emotional contagion processes, which seemed to have linked SMI images with higher social and performance-related self-esteem in a direct relationship.

Our study highlights the complex nature of online social comparison processes, demonstrating that the relationship between viewing SMI images on Instagram, social comparison and self-esteem is more intricate than expected. Our results support the idea that SMIs are a potent source of self-evaluative information, capable of evoking upward comparisons, which were previously considered as harmful. Our results align with contemporary research, highlighting the ambivalent consequences of online comparisons on self-esteem. On one hand, we provide evidence that exposure SMIs’ positive self-presentation on Instagram may not necessarily ruin viewers’ self-evaluations and self-esteem, contrary to prior research. Instead, our study shows that such images may even temporarily boost self-esteem, possibly through upward assimilation or emotional contagion processes. However, it is important to acknowledge that these upward comparison processes may also have negative consequences on individuals’ self-esteem regarding physical appearance.

### 5.2. The moderating role of resilience

Previous studies have linked upward social comparison in SNS environments to negative psychological outcomes, but few have explored protective factors (Verduyn et al., 2017). In our investigation, we examined resilience as a potential moderator in social media contexts regarding exposure to SMI content, social comparison, and state self-esteem. Although our preliminary analyses indicated positive correlations between individual resilience, social comparison, and state self-esteem, it did not emerge as a moderator. This implies that resilience may be more closely associated to an individual’s general disposition toward social comparison and self-esteem, rather than explicitly moderating these variables in social media. Considering the complex dynamics of social media interactions in our study, other moderators like self-concept could have a stronger impact on responses to SMI content (Carter and Vartanian, 2022). Carter and Vartanian (2022) highlight the role of self-concept clarity in moderating the connection between exposure to thin-ideal images and body dissatisfaction through appearance-social comparison, suggesting that individuals with a less defined self-concept tend to engage in social comparisons to understand their societal role. Future research should explore if other moderators can explain the effects of positively-biased SMI images.

### 5.3. Limitations and implications

One major limitation of this study is the small sample size, potentially impacting statistical power and limiting the detection of significant effects in the moderated mediation analysis of resilience. A larger sample size would enable a deeper investigation of the suppression effect and enhance the detection of both direct and indirect effects. Furthermore, the sample only included female students, justified by research indicating gender differences in social media impacts. While our findings provide insights into women’s experiences, their generalizability to other genders remains limited. Future studies should employ a more diverse sample of various demographic and socioeconomic groups. It’s worth noting that our experimental manipulation of image type through a questionnaire may not fully replicate the experience of scrolling through Instagram, lacking crucial features like access to comments. As users spend more time on social media (DataReportal, 2022), the observed effects might be more pronounced in the real app usage. Another limitation lies in our use of the Allan and Gilbert (1995) scale for social comparison, which conflates comparison direction with comparison frequency due to its semantic differential scale and item wording. Moreover, the scale mixes items related to both vertical and horizontal dimensions, complicating result interpretation. Given its publication year, like the self-esteem measure, it lacks validation for online settings, raising concerns about reliability and validity. Future studies should prioritize developing suitable measures for assessing social comparison in social media contexts. Lastly, our study exclusively featured SMI profiles with large audiences and similar content types. Prior research suggests that SMIs with smaller audiences are perceived as more authentic and less relatable as their popularity grows (Ruiz-Gómez, 2019). Future studies should incorporate SMIs with varying audience size to explore their impact on perceived similarity and identification levels.

## 6. Conclusion

In conclusion, this study offers new insights into the impact of social media on psychological well-being by investigating the relationships between exposure to positivity-biased images of SMIs, social comparison, state self-esteem, and resilience. Our findings revealed a more complex web of relationships than expected, highlighting both potential risks and unexpected self-esteem benefits. We uncovered a suppression effect, possibly due to simultaneous contrastive/assimilative comparisons and emotional contagion mechanisms, with distinct effects on self-esteem dimensions. Although individual resilience correlated with higher state self-esteem and positive self-evaluation in social comparison, it did not moderate the influence of SMI images on psychological outcomes. Our findings underscore the importance of promoting digital literacy and emotional well-being in a society deeply affected by social media, guiding individuals in mindful online interactions. This study aligns with recent research challenging the notion that social media and influencers inherently harm mental well-being, emphasizing the necessity for future research into the intricate interplay of psychological variables within social media environments.

## Data availability statement

The raw data supporting the conclusions of this article will be made available by the authors, without undue reservation.

## Ethics statement

The present study was conducted in full accordance with the Ethical Guidelines of the German Association of Psychologists and the American Psychological Association. Ethical approval was not required for this study at the respective university. However, the framework of this study was ethically approved and exclusively makes use of anonymous questionnaires. We had no reasons to assume that our survey would induce persistent negative psychological states in the participants.

## Author contributions

All authors developed the study concept and contributed to the study design. LR collected and analyzed the data and wrote the manuscript draft. JJ and TM supervised the study and revised the manuscript draft. All authors approved the final version to be published and agree to take responsibility and be held accountable for the integrity of the data and accuracy of the data analysis.

## Footnotes

^1^We chose not to measure actual body weight because research (e.g., Ali et al., 2010) suggests a strongly negative and significant relationship between *self-perceived* weight status and mental health, whereas there is no clear connection between *actual* weight status and mental health outcomes.

## References

[ref1] Ali M. M. Fang H. Rizzo J. A. (2010). Body weight, self-perception and mental health outcomes among adolescents. J. Ment. Health Policy Econ. 13, 53–63. PMID: 20919592

[ref2] Allan S. Gilbert P. (1995). A social comparison scale: psychometric properties and relationship to psychology. Personal. Individ. Differ. 19, 293–299. doi: 10.1016/0191-8869(95)00086-L

[ref3] Appel M. Marker C. Gnambs T. (2020). Are social media ruining our lives? A review of Meta-analytical evidence. Rev. Gen. Psychol. 24, 60–74. doi: 10.1177/1089268019880891

[ref4] Bell B. T. (2019). “You take fifty photos, delete forty-nine and use one”: a qualitative study of adolescent image-sharing practices on social media. Int. J. Child Comp. Interact. 20, 64–71. doi: 10.1016/j.ijcci.2019.03.002

[ref5] Benetti C. Kambouropoulos N. (2006). Affect-regulated indirect effects of trait anxiety and trait resilience on self-esteem. Personal. Individ. Differ. 41, 341–352. doi: 10.1016/j.paid.2006.01.015

[ref6] Bilgin O. Taş I. (2018). Effects of perceived social support and psychological resilience on social media addiction among university students. Univ. J. Educ. Res. 6, 751–758. doi: 10.13189/ujer.2018.060418

[ref7] Bleidorn W. Arslan R. C. Denissen J. J. A. Rentfrow P. J. Gebauer J. E. Potter J.. (2016). Age and gender differences in self-esteem – a cross-cultural window. J. Pers. Soc. Psychol. 111, 396–410. doi: 10.1037/pspp0000078 26692356

[ref9] Carter J. J. Vartanian L. R. (2022). Self-concept and appearance-based social comparison to idealized bodies. Body Image 40, 124–130. doi: 10.1016/j.bodyim.2021.12.001, PMID: 34929487

[ref10] Chae J. (2018). Explaining females’ envy towards social media influencers. Media Psychol. 21, 246–262. doi: 10.1080/15213269.2017.1328312

[ref11] Choi S. Kim E. (2021). Between Instagram browsing and subjective well-being: social comparison or emotional contagion? Media Psychol. 24, 866–890. doi: 10.1080/15213269.2020.1824120

[ref12] Collins R. L. (2000). “Among the better ones: upward assimilation in social comparison” in Handbook of social comparison: Theory and research. eds. Suls J. Wheeler L. (Dordrecht: Kluwer Academic Publishers)

[ref13] DataReportal. (2022). Ranking der Länder mit höchster durchschnittlicher Nutzungsdauer von Social Networks weltweit im Jahr 2021 (in Minuten pro Tag) [Infographic]. Statista. Available at: https://de.statista.com/statistik/daten/studie/160137/umfrage/verweildauer-auf-social-networks-pro-tag-nach-laendern/ https://de.statista.com/statistik/daten/studie/160137/umfrage/

[ref14] de Vries D. A. Möller A. M. Wieringa M. S. Eigenraam A. W. Hamelink K. (2017). Social comparison as the thief of joy: emotional consequences of viewing strangers’ Instagram posts. Media Psychol. 21, 222–245. doi: 10.1080/15213269.2016.1267647

[ref15] Döring N. Bortz J. (2016). Forschungsmethoden und evaluation in den Sozial- und Humanwissenschaften [research methods and evaluation in the social and human sciences]. 5th London: Springer.

[ref16] Festinger L. (1954). A theory of social comparison processes. Hum. Relat. 7, 117–140. doi: 10.1177/001872675400700202

[ref17] Field A. (2018). Discovering statistics using IBM SPSS statistics (5th). London: SAGE.

[ref19] Gonzales A. L. Hancock J. T. (2011). Mirror, mirror on my Facebook wall: effects of exposure to Facebook on self-esteem. Cyberpsychol. Behav. Soc. Netw. 14, 79–83. doi: 10.1089/cyber.2009.0411, PMID: 21329447

[ref20] Gräve J.-F. (2017). Exploring the perception of influencers vs. traditional celebrities: are social media stars a new type of endorser? Proceedings of the 8th international conference on Social Media and Society, Association for Computing Machinery, 36, 1–5.

[ref21] Grogan S. Williams Z. Conner M. (1996). The effects of viewing same-gender photographic models on body-esteem. Psychol. Women Q. 20, 569–575. doi: 10.1111/j.1471-6402.1996.tb00322.x

[ref22] Hajli M. N. (2014). A study of the impact of social media on consumers. Int. J. Mark. Res. 56, 387–404. doi: 10.2501/IJMR-2014-025

[ref23] Hayes A. F. (2022). *Introduction to mediation, moderation, and conditional process analysis: a regression- based approach*. 3rd edn. Taylor & Francis.

[ref24] Heatherton T. F. Polivy J. (1991). Development and validation of a scale for measuring state self-esteem. J. Pers. Soc. Psychol. 60, 895–910. doi: 10.1037/0022-3514.60.6.895

[ref25] Herring S. C. Kapidzic S. (2015). “Teens, gender, and self-presentation in social media” in International encyclopedia of social and behavioral sciences. ed. Wright J. D. (Oxford, England: Elsevier)

[ref26] Ho S. Lee E. W. Liao Y. (2016). Social network sites, friends, and celebrities: the roles of social comparison and celebrity involvement in adolescents’ body image dissatisfaction. Soc. Media Soc. 2, 1–11. doi: 10.1177/2056305116664

[ref27] IBM Corp. (2022). IBM SPSS statistics for Macintosh (version 29.0). New York: IBM Corp.

[ref28] InfluencerDB. (2020a). Most-followed German influencers on Instagram as of December 2019 (in millions) [infographic]. Statista. Available at: https://www.statista.com/statistics/1087614/most-followers-instagram-influencers-germany/

[ref29] InfluencerDB. (2020b). Most-followed German fashion influencers on Instagram as of December 2019 (in 1,000s) [infographic]. Statista. Available at: https://www.statista.com/statistics/1047355/most-followers-instagram-influencers-fashion-germany/

[ref30] Kaiser N. Seves M. Koutsouleris N. Ruhrmann S. (2019). Validierung einer deutschen Version der Resilience Scale for Adults (RSA). Diagnostica 65, 205–215. doi: 10.1026/0012-1924/a000228

[ref31] Kim H. Schlicht R. Schardt M. Florack A. (2021). The contributions of social comparison to social network site addiction. PLoS One 16:257795. doi: 10.1371/journal.pone.0257795 PMC8553147 34710108

[ref32] Krause H.-V. Baum K. Baumann A. Krasnova H. (2021). Unifying the detrimental and beneficial effects of social network sites on self-esteem: a systematic literature review. Media Psychol. 24, 10–47. doi: 10.1080/15213269.2019

[ref1001] Leipold B. (2015). Resilienz im Erwachsenenalter [Resilience in Adulthood]. 1st edn. Ernst Reinhardt Verlag.

[ref33] Liu D. Baumeister R. F. (2016). Social networking online and personality of self-worth: a meta-analysis. J. Res. Pers. 64, 79–89. doi: 10.1016/j.jrp.2016.06.024

[ref34] Locke K. D. (2005). Connecting the horizontal dimension of social comparison with self-worth and self-confidence. Soc. Pers. Soc. Psychol. 31, 795–803. doi: 10.1177/0146167204271634 15833906

[ref35] Lup K. Trub L. Rosenthal L. (2015). Instagram #Instasad?: exploring associations among Instagram use, depressive symptoms, negative social comparison, and strangers followed. Cyberpsychol. Behav. Soc. Netw. 18, 247–252. doi: 10.1089/cyber.2014.0560 25965859

[ref36] MacKinnon D. P. Krull J. L. Lockwood C. M. (2000). Equivalence of mediation, confounding and suppression effect. Prev. Sci. 1, 173–181. doi: 10.1023/A:1026595011371, PMID: 11523746 PMC2819361

[ref38] Midgley C. Thai S. Lockwood P. Kovacheff C. Page-Gould E. (2021). When every day is a high school Reunion: social media comparisons and self-esteem. J. Pers. Soc. Psychol. Interpers. Relat. Group Process. 121, 285–307. doi: 10.1037/pspi0000336 32790470

[ref39] Pedalino F. Camerini A.-L. (2022). Instagram use and body dissatisfaction: the mediating role of upward social comparison with peers and influencers among young females. Int. J. Environ. Res. Public Health 19:1543. doi: 10.3390/ijerph19031543 35162562 PMC8834897

[ref40] Ruiz-Gómez A. (2019). Digital fame and fortune in the age of social media: a classification of social media influencer. Revista Internacional de Investigación en Comunicación 19, 8–29. doi: 10.7263/adresic-019-01

[ref41] Schreurs L. Meier A. Vandenbosch L. (2022). Exposure to the positivity Bias and Adolescents' differential longitudinal links with social comparison, inspiration and envy depending on social media literacy. Curr. Psychol. 21, 1–21. doi: 10.1007/s12144-022-03893-3, PMID: 36373115 PMC9638310

[ref42] Schreurs L. Vandenbosch L. (2021). Introducing the social media literacy (SMILE) model with the case of the positivity bias on social media. J. Child. Media 15, 320–337. doi: 10.1080/17482798.2020.1809481

[ref44] Tiggeman M. Zaccardo M. (2015). “Exercise to be fit, not skinny”: the effect of fitspiration imagery on women’s body image. Body Image 15, 61–67. doi: 10.1016/j.bodyim.2015.06.003, PMID: 26176993

[ref45] Valls M. (2022). Gender differences in social comparison processes and self-concept among students. Front. Educ. 6:815619. doi: 10.3389/feduc.2021.815619

[ref46] Verduyn P. Ybarra O. Résibos M. Jonides J. Kross E. (2017). Do social networking sites enhance or undermine well-being? A critical review. Soc. Issues Policy Rev. 11, 274–302. doi: 10.1111/sipr.12033

[ref47] Windle G. (2011). What is resilience? A review and concept analysis. Rev. Clin. Gerontol. 21, 152–169. doi: 10.1017/S0959259810000420

